# Single-nucleotide polymorphism rs4664308 in PLA2R1 gene is associated with the risk of idiopathic membranous nephropathy: a meta-analysis

**DOI:** 10.1038/s41598-020-70009-x

**Published:** 2020-08-04

**Authors:** Masahiro Yoshikawa, Kensuke Asaba

**Affiliations:** 1 Division of Laboratory Medicine, Department of Pathology and Microbiology, Nihon University School of Medicine, Tokyo, Japan; 2 Department of Computational Diagnostic Radiology and Preventive Medicine, The University of Tokyo Hospital, Tokyo, Japan

**Keywords:** Clinical genetics, Membranous nephropathy

## Abstract

Although many studies investigated the associations between single-nucleotide polymorphisms (SNPs) in the M-type phospholipase A2 receptor-1 (PLA2R1) gene and susceptibility to idiopathic membranous nephropathy (IMN), some showed inconsistent results. Here, we conducted a meta-analysis examining the associations between PLA2R1 SNPs and IMN susceptibility after systematic searches in the PubMed and Web of Science databases. Our meta-analysis for rs4664308 A>G including 2,542 IMN patients and 4,396 controls in seven studies showed a significant association between the G allele and a lower risk of IMN, as determined using an allelic model (odds ratio, 0.45; 95% confidence interval [0.41–0.50]), an additive model (for GG vs. AA: 0.26; [0.21–0.33]; for AG vs. AA: 0.40; [0.36–0.45]), a dominant model (0.37; [0.34–0.42]) and a recessive model (0.38; [0.31–0.48]). Our meta-analysis also suggested associations between rs3828323, rs35771982, rs3749117 and rs3749119 and IMN susceptibility although high heterogeneities and/or publication biases were observed. We did not study in our meta-analysis, but other studies indicated that high-risk genotype combinations of rs2187668 in the human leucocyte antigen-DQ a-chain 1 gene and rs4664308 in the PLA2R1 gene had even stronger associations and could affect the formation of anti-PLA2R1 antibodies, suggesting these SNPs could be novel therapeutic targets.

## Introduction

Membranous nephropathy (MN) is a type of glomerulonephritis in which a thickening of the glomerular capillary walls is observed because of subepithelial depositions or the in situ formations of immune complexes on the outer aspect of the glomerular basement membrane^1–3^. The incidence of MN is approximately 1 case per 100,000 persons per year^4^, and MN is one of the most common causes of adult onset nephrotic syndromes. Actually, about 20% of nephrotic syndrome cases have MN^1^. MN is classified into primary or idiopathic MN (IMN) or secondary MN (SMN), and nearly 70% to 80% of MN cases have IMN^1,2^. SMN is often caused by autoimmune diseases, malignant diseases, infectious diseases and medications^5^. Most cases of IMN undergo remission spontaneously or with immunosuppressive therapies. On the other hand, renal function gradually declines in refractory cases of IMN (about 30% of IMN cases), and renal replacement therapies may be necessary within a few decades of life^1,5,6^. Therefore, it is important to elucidate the pathology in more detail and to identify new therapeutic targets.

IMN is considered to be an organ-specific type of autoimmune glomerulonephritis, but the pathophysiology is not fully understood. In 2009, Beck et al. reported that M-type phospholipase A2 receptor-1 (PLA2R1) protein and anti-PLA2R1 auto-antibodies (auto-Abs) (mainly immunoglobulin [Ig]G4 isotype) were co-localized and deposited in podocytes in a majority (about 70%) of IMN cases, but not in patients with SMN, lupus nephritis or IgA nephropathy^7^. In 2011, Stanescu et al. performed genome wide association studies (GWASs) and found that two single-nucleotide polymorphisms (SNPs), rs2187668 and rs4664308, in the genes encoding human leucocyte antigen-DQ a-chain 1 (HLA-DQA1) and PLA2R1, respectively, were significantly associated with the risk of IMN in patients of white ancestry^8^. Recently, several case–control studies have been performed to investigate the associations between SNPs in the genes encoding HLA-DQA1 and/or PLA2R1 and IMN susceptibility. In 2018, Bao et al. performed a meta-analysis to confirm that HLA-DQA1 rs2187668 was associated with the risk of IMN^9^. However, a meta-analysis investigating the associations between SNPs in the PLA2R1 gene and IMN susceptibility has not yet been performed. Moreover, some case–control studies examining associations between SNPs in the PLA2R1 gene and IMN susceptibility have shown inconsistent results. Therefore, we performed the present meta-analysis to investigate the associations between SNPs in the PLA2R1 gene and IMN susceptibility, and discussed the pathophysiology of IMN.

## Results

### Database search and profiles of studies

**Figure 1:**
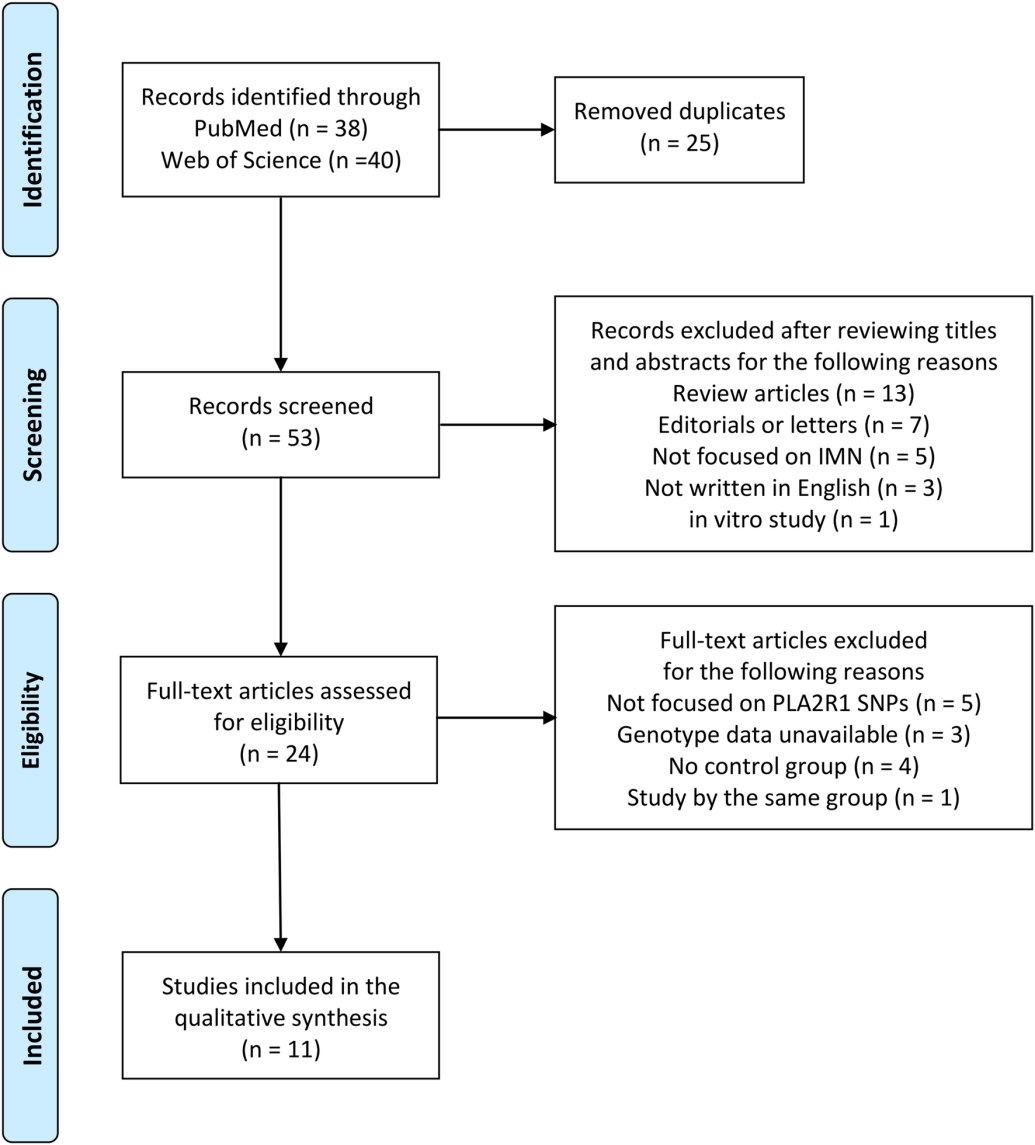
Flow diagram^21^ for our search strategy and process.

We searched for studies in the PubMed and Web of Science databases and identified a total of 78 articles. After removing 25 duplicates, we reviewed the titles and/or abstracts and excluded 29 articles. We then assessed the full texts of the remaining 24 articles and excluded 13 more articles. Among the remaining 24 articles, we noticed that the studies by Cui et al.^10^ and Lv et al.^11^ were conducted by the same group. We compared these two studies carefully, but could not rule out the possibility that the study populations were duplicated. Therefore, we decided to adopt the study by Lv et al.^11^ with the larger study population and exclude the study by Cui et al.^10^. Finally, we adopted 11 articles in which the associations between SNPs in the PLA2R1 gene (rs4664308, rs3828323, rs35771982, rs3749117, rs3749119, rs2715918, rs4665143, rs6757188 and rs17830558) and IMN susceptibility had been investigated^8,11–20^. A flow diagram showing our search strategy and process is presented in Fig. 1^21^. However, rs2715918, rs4665143, rs6757188 and rs17830558 were investigated in just one or two studies. Therefore these four SNPs were excluded from our meta-analysis (as a result, a study by Sekula et al.^20^ was not included in our meta-analysis).

### A meta-analysis for rs4664308 A>G

**Figure 2:**
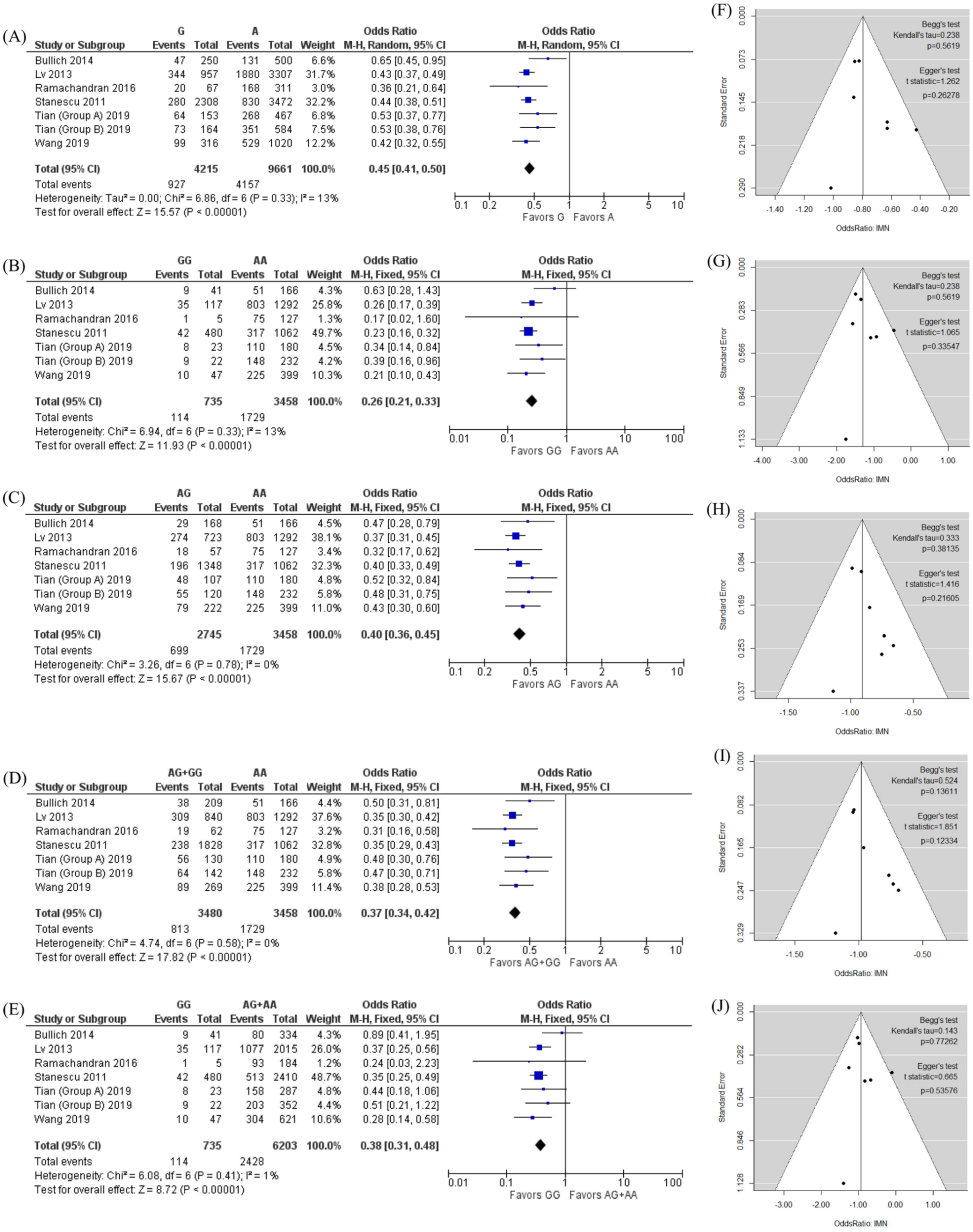
Forest plot of the risk of IMN associated with rs4664308 under the (**A**) allelic model (G vs A), (**B**) additive model (GG vs. AA), (**C**) additive model (AG vs. AA), (**D**) dominant model (AG + GG vs. AA), and (**E**) recessive model (GG vs. AG + AA). (**F**–**J**) Funnel plot, Begg’s test and Egger’s test for (**A**–**E**), respectively. Note that the log (OR) is plotted on the horizontal axis.

First, we conducted a meta-analysis to determine the associations between rs4664308 and IMN susceptibility. A total of seven studies (by six groups) were included with a total of 2,542 IMN patients and 4,396 controls^8,11–15^. The profiles of the seven studies are shown in Table 1. The control groups in all seven included studies were in accordance with Hardy–Weinberg equilibrium (HWE)^22^ (*P* > 0.05). The qualities of all seven included studies were judged to be high (Newcastle–Ottawa scale [NOS]^23^ score ≥ 7). After we extracted the data on the genotype distributions from each study, we conducted a meta-analysis by combining the odds ratio (OR) for the risk of development of IMN in each study. We observed statistically significant differences with low heterogeneities under an allelic model (G vs. A: OR, 0.45; 95% confidence interval [CI] 0.41–0.50; I^2^ = 13%), an additive model (for GG vs. AA: OR, 0.26; 95% CI 0.21–0.33; I^2^ = 13%; for AG vs. AA: OR, 0.40; 95% CI 0.36–0.45; I^2^ = 0%), a dominant model (AG + GG vs. AA: OR, 0.37; 95% CI 0.34–0.42; I^2^ = 0%) and a recessive model (GG vs. AG + AA: OR, 0.38; 95% CI 0.31–0.48, I^2^ = 1%) as shown in Fig. 2A–E. There were no publication biases as assessed using both Begg’s and Egger’s tests^24,25^ (*P* > 0.10, as shown in Fig. 2F–J).

**Table 1:** Characteristics and rs4664308 A > G genotype distributions (expressed in numbers) between the case and control groups in the included studies.

Author Year	Population or ethnicity	Genotyping	Cases/controls	Genotypes of cases				Genotypes of controls				HWE for controls	NOS
				AA	AG	GG	MAF	AA	AG	GG	MAF		
Bullich 2014	Spanish	Applied biosystems	89/286	51	29	9	0.264	115	139	32	0.355	0.299	8
Lv 2013	Han	Applied biosystems	1,112/1,020	803	274	35	0.155	489	449	82	0.300	0.132	7
Ramachandran 2016	Indian	Applied biosystems	94/95	75	18	1	0.106	52	39	4	0.247	0.318	7
Stanescu 2011	French, Dutch and British	Illumina	555/2,335	317	196	42	0.252	745	1,152	438	0.434	0.844	7
Tian—Group A 2019	Han in Southern China	Applied biosystems	166/144	110	48	8	0.193	70	59	15	0.309	0.626	8
Tian—Group B 2019	Han in North- western China	Applied biosystems	212/162	148	55	9	0.172	84	65	13	0.281	0.932	8
Wang 2019	Han in Western China	Applied biosystems	314/354	225	79	10	0.158	174	143	37	0.306	0.349	8

*HWE* Hardy–Weinberg equilibrium, *MAF* minor allele frequency, *NOS* Newcastle–Ottawa scale.

### Meta-analyses for other SNPs

Next, we conducted a meta-analysis to determine the associations between rs3828323, rs35771982, rs3749117 and rs3749119 and IMN susceptibility. The profiles of each of the included studies are shown in Supplementary Tables S1–S4 online. Some studies were excluded because the control groups were not in accordance with HWE (*P* < 0.05). For rs3828323, we observed statistically significant differences without heterogeneities under an allelic model (T vs. C: OR, 0.68; 95% CI 0.58–0.80; I^2^ = 0%), an additive model (for TT vs. CC: OR, 0.58; 95% CI 0.39–0.84; I^2^ = 0%; for CT vs. CC: OR, 0.64; 95% CI 0.52–0.79; I^2^ = 0%), a dominant model (CT + TT vs. CC: OR, 0.63; 95% CI 0.51–0.76, I^2^ = 0%) and a recessive model (TT vs. CT + TT: OR, 0.66; 95% CI 0.46–0.96; I^2^ = 0%) in the five included studies with a total of 844 IMN patients and 805 controls^13–16^ (Supplementary Fig. S1A–E online). However, there were publication biases in additive and recessive models as assessed using both Begg’s and Egger’s tests (*P* < 0.10, as shown in Supplementary Fig. S1F–J on line). For rs35771982, we observed statistically significant differences under an additive model (CG vs. GG: OR, 0.47; 95% CI 0.35–0.62; I^2^ = 63%) and a dominant model (CG + CC vs. GG: OR, 0.53; 95% CI 0.36–0.77, I^2^ = 82%) in the eight included studies with a total of 2,438 IMN patients and 2,350 controls^11,13–15,17–19^ (Supplementary Fig. S2A–E online), although high heterogeneities and publication bias were also observed (Supplementary Fig. S2F–J on line). For rs3749117, we observed statistically significant differences under an allelic model (C vs. T: OR, 0.64,95% CI 0.43–0.96, I^2^ = 87%), an additive model (CC vs. TT: OR, 0.43; 95% CI 0.25–0.74; I^2^ = 62%) and a recessive model (CC vs. CT + TT: OR,0.46; 95% CI 0.34–0.61; I^2^ = 0%) in the four included studies with a total of 1,589 IMN patients and 1,421 controls^11,13,14^ (Supplementary Fig. S3A–E online), although high heterogeneity and publication biases were also observed (Supplementary Fig. S3F–J online). For rs3749119, we observed statistically significant differences without heterogeneities under an allelic model (T vs. C: OR, 0.75, 95% CI 0.60–0.94, I^2^ = 0%), an additive model (CT vs. CC: OR, 0.71; 95% CI 0.53–0.95; I^2^ = 0%) and a dominant model (CT + TT vs. CC: OR, 0.69; 95% CI 0.52–0.92, I^2^ = 0%) in the three included studies with a total of 436 IMN patients and 356 controls^14,16^ (Supplementary Fig. S4A–E online), although a publication bias was also observed (Supplementary Fig. S4E,J online).

## Discussion

IMN is an organ-specific type of autoimmune glomerulonephritis. Beck et al. showed that PLA2R1 protein and anti-PLA2R1 auto-Abs were co-localized and deposited in podocytes in about 70% of IMN cases^7^. Stanescu et al. showed that rs2187668 in the HLA-DQA1 gene on chromosome 6p21 and rs4664308 in the PLA2R1 gene on chromosome 2q23-24 were significantly associated with the susceptibility to IMN using GWASs^8^. These results suggested that genetic variations of HLA-DQA1 and PLA2R1 proteins might facilitate auto-Ab formation against antigens at podocytes during the development of IMN. Since then, several studies have reported associations between HLA-DQA1 SNP rs2187668 and IMN susceptibility, and a recent meta-analysis indicated that the minor A allele was associated with IMN susceptibility, though some heterogeneities were observed^9^. As for PLA2R1 SNPs, not only rs4664308, but also other SNPs were reported to be associated with IMN susceptibility in numerous studies, although some studies showed inconsistent results. To the best of our knowledge, the present study is the first meta-analysis to investigate the associations between PLA2R1 SNPs and IMN susceptibility. Our meta-analysis indicated that rs4664308 A (the major allele) was a risk allele associated with IMN susceptibility without high heterogeneities or publication biases. On the other hand, determining whether rs3828323, rs35771982, rs3749119 or rs3749117 are associated with IMN susceptibility might not be appropriate because of the high heterogeneities, publication biases, and/or limited number of studies.

Some studies have shown contradictory results regarding the associations between IMN susceptibility and a few SNPs, especially rs35771982 in the PLA2R1 gene, probably because there are genetic heterogeneities among different populations. Although we could not include the study by Zhou et al. because it was not published in English, they reported that the CC genotype and C allele in rs35771982 were common among IMN patients, compared with a normal control group, in the Chinese Han population^26^. This finding was consistent with the results of a study by Tian et.al.^14^ but was inconsistent with the results of a study by Wang et al.^15^, as shown in the Supplementary Table S2 online. Wang et al. reported that genetic heterogeneities may exist even among Chinese residents because of the diversity in geographical populations in China^15^.

Both rs2187668 alone and rs4664308 alone were associated with IMN susceptibility, as shown by the past^9^ and the present meta-analysis. We have not studied SNPs in the HLA-DQA1 gene or interactions between the HLA-DQA1 and PLA2R1 genes in the present meta-analysis, but other researchers have reported that high-risk genotype combinations of both rs2187668 and rs4664308 have even stronger associations. Stanescu et al. showed that the genotype combination of rs2187668 AA and rs4664308 AA had a much higher risk for the development of IMN than that of rs2187668 GG and rs4664308 GG (OR, 78.46; 95% CI 34.55–178.17)^8^. Ramachandran et al. showed that the same combination as that reported by Stanescu et al. had a 58.33-folds higher risk (95% CI 7.30–567.30)^13^. Lv et al. showed that the genotype combination of rs2187668 AA or AG and rs4664308 AA had a 11.13-fold higher risk than that of rs2187668 GG and rs4664308 GG (95% CI 6.47–19.15)^11^. Bullich et al. showed that the genotype combination of rs2187668 AA or AG and rs4664308 AA had a 7.33-fold higher risk than that of rs2187668 GG and rs4664308 GG or AG (95% CI 3.55–15.13)^12^. As for other SNPs, Sekula et al. showed that the genotype combination of rs9272729 AA in the HLA-DQA1 gene and rs17830558 GT in the PLA2R1 gene had a 79.4-fold higher risk than that of rs9272729 GG and rs17830558 GG (95% CI 9.17–686.85)^20^. Thiri et al. showed that the interaction between the HLA-DRB1*15:01–HLA-DQB1*06:02 haplotype and a novel SNP rs2715928 AA in the PLA2R1 gene was strongly associated with IMN in the Japanese population (OR, 15.91; 95% CI 8.94–28.3)^27^. It remains unclear how these combinations of HLA-DQA1 and PLA2R1 SNPs are associated with IMN susceptibility. Gupta et al. supposed that these SNPs might affect anti-PLA2R1 Ab production by controlling the fragmentation pattern of the antigen (PLA2R1) that is presented to T-cells on the class II receptor groove (HLA-DQA1) of antigen-presenting cells^28^. Consistent with their hypothesis, Lv et al. showed that anti-PLA2R1 Abs were detected in 19 of the 26 (73%) IMN patients with both HLA-DQA1 and PLA2R1 high-risk genotypes (AA or AG in rs2187668, AA in rs4664308, GG in rs35771982 and TT in rs3749117) while anti-PLA2R1 Abs were not detected in any of the 19 (0%) patients with both PLA2R1 and HLA-DQA1 low-risk genotypes (GG in rs2187668, GG in rs4664308, CC in rs35771982 and CC in rs3749117)^11^. Moreover, a meta-analysis including 23 studies showed that IMN patients who tested negative for anti-PLA2R1 Ab at the time of renal biopsy had a higher risk ratio (RR) of clinical remission (defined as proteinuria < 3.5 g/day and > 50% reduction from baseline proteinuria) than those who tested positive for anti-PLA2R1 Ab (RR, 1.31; 95% CI 1.12–1.46; I^2^ = 74%)^29^. Another meta-analysis including eleven studies also showed that the anti-PLA2R1 Ab-positive group had a lower RR of clinical remission (defined as proteinuria < 3.5 g/day) than the anti-PLA2R1 Ab-negative group among IMN patients (RR, 0.76; 95% CI 0.68–0.86; I^2^ = 39%)^30^. In addition, Seitz-Polski et al. showed that analysis of PLA2R1 epitope spreading was also a powerful tool for predicting clinical remission (defined as a urinary protein/creatinine ratio < 4 g/g and an estimated glomerular filtration rate > 45 ml/min/1.73 m^2^) in a cohort with 69 IMN patients who tested positive for anti-PLA2R1 Ab^31^. In their study, 20 of the 23 (87%) patients with anti-PLA2R1 Ab against an outer epitope (cysteine-rich domain) alone achieved clinical remission. On the other hand, seven of the 14 (only 50%) patients and 12 of the 32 (only 38%) patients, with anti-PLA2R1 Ab against an inner epitope (C-type lectin domain 1) and with anti-PLA2R1 Abs against two inner epitopes (C-type lectin domain 1 and C-type lectin domain 7), respectively, achieved clinical remission.

Even if the IMN risk increases by 10 to 100-fold as a result of combinations of risk alleles in HLA-DQA1 and PLA2R1 SNPs, IMN remains a rare disease with an incidence of about one patient per 100,000 persons per year^4^. Considering that some risk alleles in PLA2R1 SNPs (e.g., the A allele in rs4664308) are common, the development of IMN can not be explained by these SNPs alone. Coenen et al. speculated that a combination of both HLA-DQA1 and PLA2R1 SNPs might result in rare haploblocks conferring IMN susceptibility^32^. Moreover, other triggers, including environmental factors such as heavy-metals and drugs, are also important in the development of IMN^28,32^. Regardless, the genotype combination of HLA-DQA1 and PLA2R1 SNPs can actually increase the production of anti-PLA2R1 Abs and IMN susceptibility as shown by Lv et al.^11^. Furthermore, Bullich et al. reported that the high-risk genotype combination of AA or AG in rs2187668 and AA in rs4664308 predicted a better response to immunosuppressive therapy against IMN^12^. These results are important for understanding the pathophysiology of IMN in more detail and for investigating new therapeutic targets.

In addition to SNPs in the HLA-DQA1 and PLA2R1 genes, several other SNPs and auto-Abs are considered to be associated with IMN pathophisiology. Associations between SNPs in the interleukin-6 and toll-like receptor 9 genes and IMN susceptibility were reported in the Taiwanese population^33,34^. However, whether these SNPs are associated with IMN susceptibility in other populations remains unclear. Auto-Ab against thrombospondin type-1 domain-containing 7A (THSD7A) protein on podocyte membrane was identified in serum samples from anti-PLA2R1-negative IMN patients in 2014^35^. Although a study investigating the associations between SNPs in the THSD7A gene and IMN susceptibility has not yet been performed^6^, Zaghrini et al. reported that the serum anti-THSD7A Ab titers appeared to be correlated with disease activity and with clinical outcome in a cohort with 49 MN patients who tested positive for anti-THSD7A Ab^36^. Moreover, several intracellular enzymes such as aldose reductase (AR), superoxide dismutase 2 (SOD2) and α-enolase were identified as target antigens in MN^37^. In a study with 26 IMN patients who tested positive for anti-AR Ab and 38 IMN patients who tested positive for anti-SOD2 Ab, Han et al. reported that both the serum anti-AR Ab and anti-SOD2 Ab titers significantly correlated with urinary protein levels but did not correlate with disease severity^38^. Further studies are warranted to investigate whether these auto-Abs can actually be relevant biomarkers for monitoring the disease in the same way as anti-PLA2R1 Ab.

Our study had some major limitations. Firstly, only the studies published in English were included in our meta-analysis. Actually we could not include the study by Zhou et al.^26^ because the article was written in Chinese. Secondly, the studies by Kaga et al.^16^ and Saeed et al.^18^ enrolled not healthy but diseased subjects as controls. However, these studies were not included in our meta-analysis for rs4664308. Thirdly, the number of studies included in our meta-analysis was relatively small. Moreover, of the seven studies included in our meta-analysis for rs4664308, four were from China. This might lead to some biases, although European and Indian studies showed almost the same tendencies as the other four Chinese studies. More studies with larger sample sizes and in more ethnic populations are necessary for further validity of the outcomes of the present meta-analysis.

In conclusion, our meta-analysis showed that rs4664308 in the PLA2R1 gene was significantly associated with the risk of IMN. Although the risk allele of rs4664308 was common, IMN remains a rare disease in which genotype combinations with rs2187668 in the HLA-DQA1 gene and environmental factors also influence the formation of auto-Abs against PLA2R1 proteins at podocytes and trigger disease onset as indicated by other researchers. To investigate the pathology of IMN further, more studies examining SNPs in the PLA2R1 gene in combination with rs2187668 in the HLA-DQA1 gene are needed across various populations.

## Materials and methods

### Search strategy and eligibility criteria

We searched for eligible studies among all papers published prior to December 31, 2019, in the PubMed and Web of Science databases, in accordance with the Preferred Reporting Items for Systematic Reviews and Meta-Analyses guidelines^39^. Two authors (M.Y. and K.A.) performed the database searches independently, and each discrepancy was discussed until a consensus was reached. We used the following terms for the database searches: ((phospholipase A2 receptor) OR PLA2R1) AND (polymorphism OR polymorphisms OR variant OR variants) AND ((membranous nephropathy) OR (membranous glomerulonephritis)). Our eligibility criteria were as follows: (1) studies that focused on the associations between SNPs in the PLA2R1 gene and IMN susceptibility,(2) studies whose subjects were divided into control groups and IMN case groups, which were diagnosed as IMN by renal biopsy,and (3) studies that provided sufficient data in the article to calculate the ORs and 95% CIs. Our exclusion criteria were as follows: (1) review articles, case reports, editorials, letters or commentaries; (2) studies not published in English; and (3) studies in which the control group was not in accordance with HWE^22^.

### Data extraction

From the shortlisted studies, we extracted the following necessary information: the first author’s name; publication year; population or ethnicity; genotyping method; SNPs in the PLA2R1 gene; number of cases and controls; and allele and genotype frequencies.

### Quality assessment of the included studies

The NOS^23^ was used to assess the quality of the studies selected for inclusion in our meta-analysis. Two authors (M.Y. and K.A.) evaluated the scores independently, and any discrepancy was discussed until a consensus was reached.

### Data analysis and statistics

HWE^22^ for each study was tested for the control group using the chi-squared test (as shown in each Table). A *P* value below 0.05 was considered to indicate a statistically significant result in the HWE test. The heterogeneity was estimated using Cochran’s Q test and the I^2^ statistic^39,40^. The meta-analyses were conducted using random-effects models when Cochran’s Q test was significant (*P* < 0.10). Otherwise, fixed-effects models were used. The heterogeneity was categorized as low if the I^2^ was 0–25%, as moderate if the I^2^ was 25–75%, or as high if the I^2^ was 75–100%^41^. The data for the ORs and their 95% CIs were pooled, and forest plots were drawn using Review Manager, version 5.3. Begg’s and Egger’s tests^24,25^ were also conducted, and a funnel plot was drawn using R software, version 3.4.0, to assess the presence of a publication bias, as described previously^42^. A *P* value of less than 0.10 was considered as indicative of a statistically significant result in both the Begg’s and Egger’s tests^24,25^.

## Supplementary information

Supplementary Information 1.

Supplementary Information 2.

## Data Availability

All data analyzed during this study are available on reasonable request to the corresponding author.
