# Supplementary material for: Single-nucleotide polymorphism rs4664308 in PLA2R1 gene is associated with the risk of idiopathic membranous nephropathy: a meta-analysis
Source: Sci Rep. 2020 Aug 4;10:13119. doi: 10.1038/s41598-020-70009-x (PMC7403323; doi:10.1038/s41598-020-70009-x)
Supplement: Supplementary file 1 — Supplementary Information 1. [file 41598_2020_70009_MOESM1_ESM.pdf]

Single-nucleotide polymorphism rs4664308 in PLA2R1 gene is associated with the risk of idiopathic membranous nephropathy: A meta-analysis.

Masahiro Yoshikawa[1,*] and Kensuke Asaba[2]

1. Division of Laboratory Medicine, Department of Pathology and Microbiology, Nihon University School of Medicine.
2. Department of Computational Diagnostic Radiology and Preventive Medicine, The University of Tokyo Hospital.
*Corresponding author; myosh-tky@umin.ac.jp

**Author Year**	**Population or ethnicity**	**Genotyping**	**cases /controls**	**Genotypes of cases**				**Genotypes of controls**				**HWE for controls**	**NOS**
				**CC**	**CT**	**TT**	**MAF**	**CC**	**CT**	**TT**	**MAF**		
Kaga 2014	Japanese	Applied Biosystems	58/50	36	18	4	0.241	23	19	8	0.350	0.244	6
Kim 2011	Korean	Applied Biosystems	132/132	84	44	4	0.195	70	45	17	0.299	**excluded** 0.031	8
Ramachandran 2016	Indian	Applied Biosystems	94/95	75	16	3	0.117	59	29	7	0.226	0.211	7
Tian - Group A 2019	Han in Southern China	Applied Biosystems	166/144	102	52	12	0.229	77	51	16	0.288	0.101	8
Tian - Group B 2019	Han in North-western China	Applied Biosystems	212/162	129	62	21	0.245	84	58	20	0.302	0.054	8
Wang 2019	Han in Western China	Applied Biosystems	314/354	219	81	14	0.174	209	125	20	0.233	0.818	8

Supplementary Table S1. **Characteristics and rs3828323 C>T genotype distributions (expressed in numbers) between the case and control groups in the included studies.** Abbreviations: HWE= Hardy–Weinberg equilibrium; MAF=minor allele frequency; NOS= Newcastle-Ottawa scale.

Author Year	Population or ethnicity	Genotyping	cases /controls	Genotypes of cases				Genotypes of controls				HWE for controls	NOS
				GG	CG	CC	MAF	GG	CG	CC	MAF		
Kaga 2018	Japanese	Applied Biosystems	58/50	35	18	5	0.241	16	15	19	0.530	**excluded** 0.005	6
Kim 2011	Korean	Applied Biosystems	132/132	9	27	96	0.830	13	52	67	0.705	0.537	8
Ramachandran 2016	Indian	Applied Biosystems	94/95	74	18	2	0.117	49	41	5	0.268	0.335	7
Saeed 2014	Caucasians	Life Technologies	279/337	160	91	28	0.263	89	166	82	0.490	0.791	6
Liu 2010	Taiwanese-Chinese	Applied Biosystems	129/106	90	37	2	0.159	58	40	8	0.264	0.763	7
Lv 2013	Han	Applied Biosystems	1112/1020	801	278	33	0.155	486	453	81	0.301	0.082	7
Tian - Group A 2019	Han in Southern China	Applied Biosystems	166/144	9	56	101	0.777	15	61	68	0.684	0.810	8
Tian - Group B 2019	Han in North-western China	Applied Biosystems	212/162	10	65	137	0.800	10	72	80	0.716	0.237	8
Wang 2019	Han in Western China	Applied Biosystems	314/354	227	78	9	0.153	178	140	36	0.299	0.280	8

Supplementary Table S2. Characteristics and rs35771982 G>C genotype distributions (expressed in numbers) between the case and control groups in the included studies. Abbreviations: HWE= Hardy–Weinberg equilibrium; MAF=minor allele frequency; NOS=Newcastle-Ottawa scale.

**Author Year**	**Population or ethnicity**	**Genotyping**	**cases /controls**	**Genotypes of cases**				**Genotypes of controls**				**HWE for controls**	**NOS**
				**TT**	**CT**	**CC**	**MAF**	**TT**	**CT**	**CC**	**MAF**		
Kaga 2014	Japanese	Applied Biosystems	58/50	31	22	5	0.276	15	16	19	0.540	**excluded** 0.012	6
Lv 2013	Han	Applied Biosystems	1112/1020	801	276	35	0.156	489	451	80	0.300	0.086	7
Ramachandran 2016	Indian	Applied Biosystems	94/95	32	51	11	0.388	43	38	14	0.347	0.251	7
Tian - Group A 2019	Han in Southern China	Applied Biosystems	166/144	95	59	12	0.250	65	57	22	0.351	0.116	8
Tian - Group B 2019	Han in North-western China	Applied Biosystems	217/162	134	61	17	0.224	77	64	21	0.327	0.191	8

Supplementary Table S3. Characteristics and rs3749117 T>C genotype distributions (expressed in numbers) between the case and control groups in the included studies. Abbreviations: HWE= Hardy–Weinberg equilibrium; MAF=minor allele frequency; NOS= Newcastle-Ottawa scale.

Author Year	Population or ethnicity	Genotyping	cases /controls	Genotypes of cases				Genotypes of controls				HWE for controls	NOS
				**CC**	**CT**	**TT**	**MAF**	**CC**	**CT**	**TT**	**MAF**		
Kaga 2014	Japanese	Applied Biosystems	58/50	29	26	3	0.276	15	29	6	0.410	0.160	6
Ramachandran 2016	Indian	Applied Biosystems	94/95	80	6	8	0.117	55	21	19	0.311	**excluded** <0.001	7
Tian - Group A 2019	Han in Southern China	Applied Biosystems	166/144	86	66	14	0.283	65	62	17	0.333	0.708	8
Tian - Group B 2019	Han in North-western China	Applied Biosystems	212/162	130	70	12	0.222	87	65	10	0.262	0.641	8
Wang 2019	Han in Western China	Applied Biosystems	314/354	232	73	9	0.145	181	133	40	0.301	**excluded** 0.044	8

Supplementary Table S4. Characteristics and rs3749119 C>T genotype distributions (expressed in numbers) between the case and control groups in the included studies. Abbreviations: HWE= Hardy–Weinberg equilibrium; MAF=minor allele frequency; NOS= Newcast
