# Supplementary material for: Single-nucleotide polymorphism rs4664308 in PLA2R1 gene is associated with the risk of idiopathic membranous nephropathy: a meta-analysis
Source: Sci Rep. 2020 Aug 4;10:13119. doi: 10.1038/s41598-020-70009-x (PMC7403323; doi:10.1038/s41598-020-70009-x)
Supplement: Supplementary file 2 — Supplementary Information 2. [file 41598_2020_70009_MOESM2_ESM.pdf]

Single-nucleotide polymorphism rs4664308 in PLA2R1 gene is associated with the risk of idiopathic membranous nephropathy: A meta-analysis.

Masahiro Yoshikawa[1,*] and Kensuke Asaba[2]

1. Division of Laboratory Medicine, Department of Pathology and Microbiology, Nihon University School of Medicine.

2. Department of Computational Diagnostic Radiology and Preventive Medicine, The University of Tokyo Hospital.

*Corresponding author; myosh-tky@umin.ac.jp

(A)

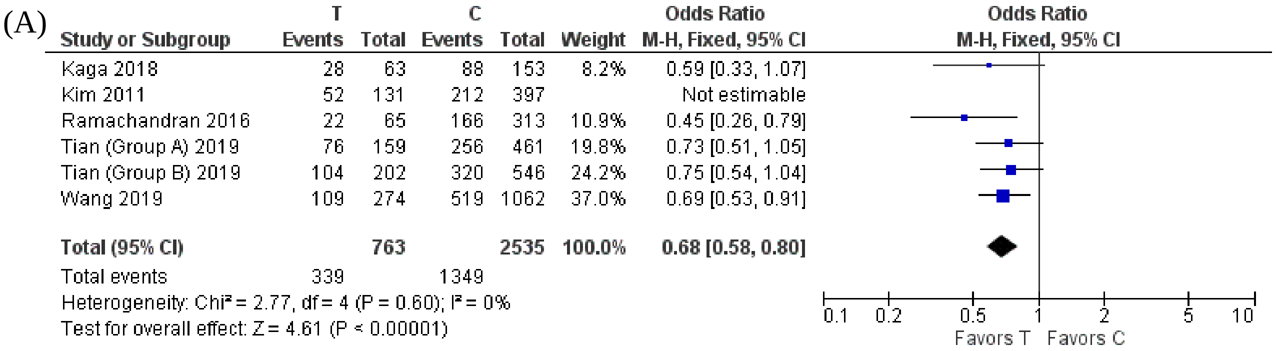

Study or Subgroup	T Events	T Total	C Events	C Total	Weight	Odds Ratio M-H, Fixed, 95% CI
Kaga 2018	28	63	88	153	8.2%	0.59 [0.33, 1.07]
Kim 2011	52	131	212	397		Not estimable
Ramachandran 2016	22	65	166	313	10.9%	0.45 [0.26, 0.79]
Tian (Group A) 2019	76	159	256	461	19.8%	0.73 [0.51, 1.05]
Tian (Group B) 2019	104	202	320	546	24.2%	0.75 [0.54, 1.04]
Wang 2019	109	274	519	1062	37.0%	0.69 [0.53, 0.91]
**Total (95% CI)**		**763**		**2535**	**100.0%**	**0.68 [0.58, 0.80]**
Total events	339		1349			

Heterogeneity: Chi² = 2.77, df = 4 (P = 0.60); I² = 0%
Test for overall effect: Z = 4.61 (P < 0.00001)

(F)

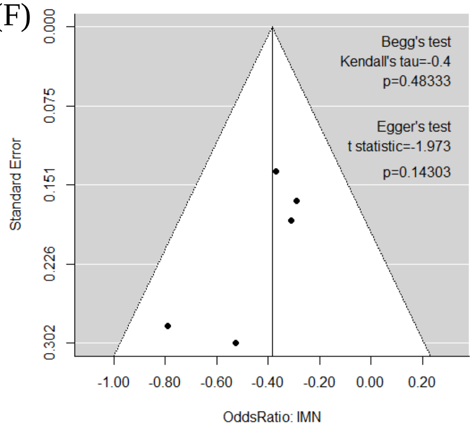

(B)

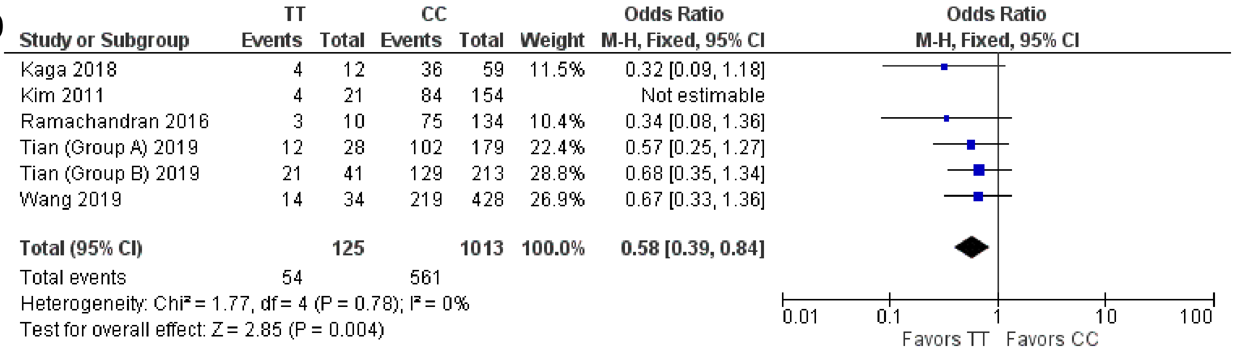

Study or Subgroup	TT Events	TT Total	CC Events	CC Total	Weight	Odds Ratio M-H, Fixed, 95% CI
Kaga 2018	4	12	36	59	11.5%	0.32 [0.09, 1.18]
Kim 2011	4	21	84	154		Not estimable
Ramachandran 2016	3	10	75	134	10.4%	0.34 [0.08, 1.36]
Tian (Group A) 2019	12	28	102	179	22.4%	0.57 [0.25, 1.27]
Tian (Group B) 2019	21	41	129	213	28.8%	0.68 [0.35, 1.34]
Wang 2019	14	34	219	428	26.9%	0.67 [0.33, 1.36]
**Total (95% CI)**		**125**		**1013**	**100.0%**	**0.58 [0.39, 0.84]**
Total events	54		561			

Heterogeneity: Chi² = 1.77, df = 4 (P = 0.78); I² = 0%
Test for overall effect: Z = 2.85 (P = 0.004)

(G)

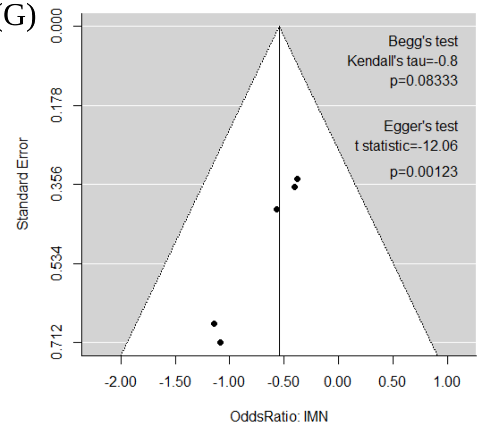

(C)

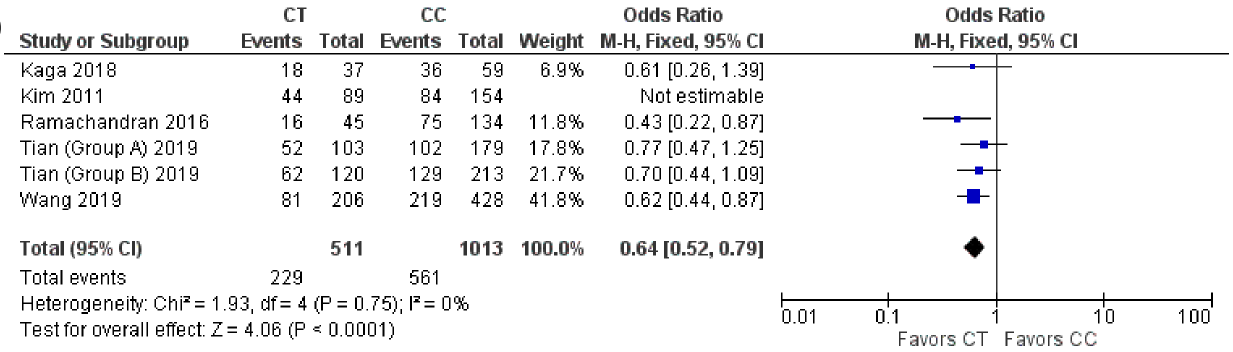

Study or Subgroup	CT Events	CT Total	CC Events	CC Total	Weight	Odds Ratio M-H, Fixed, 95% CI
Kaga 2018	18	37	36	59	6.9%	0.61 [0.26, 1.39]
Kim 2011	44	89	84	154		Not estimable
Ramachandran 2016	16	45	75	134	11.8%	0.43 [0.22, 0.87]
Tian (Group A) 2019	52	103	102	179	17.8%	0.77 [0.47, 1.25]
Tian (Group B) 2019	62	120	129	213	21.7%	0.70 [0.44, 1.09]
Wang 2019	81	206	219	428	41.8%	0.62 [0.44, 0.87]
**Total (95% CI)**		**511**		**1013**	**100.0%**	**0.64 [0.52, 0.79]**
Total events	229		561			

Heterogeneity: Chi² = 1.93, df = 4 (P = 0.75); I² = 0%
Test for overall effect: Z = 4.06 (P < 0.0001)

(H)

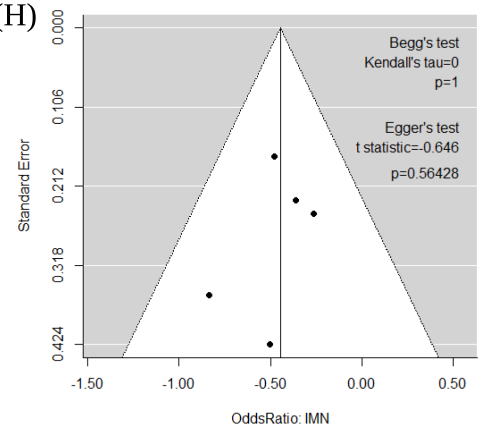

Supplementary Figure S1 (continued)

(D)

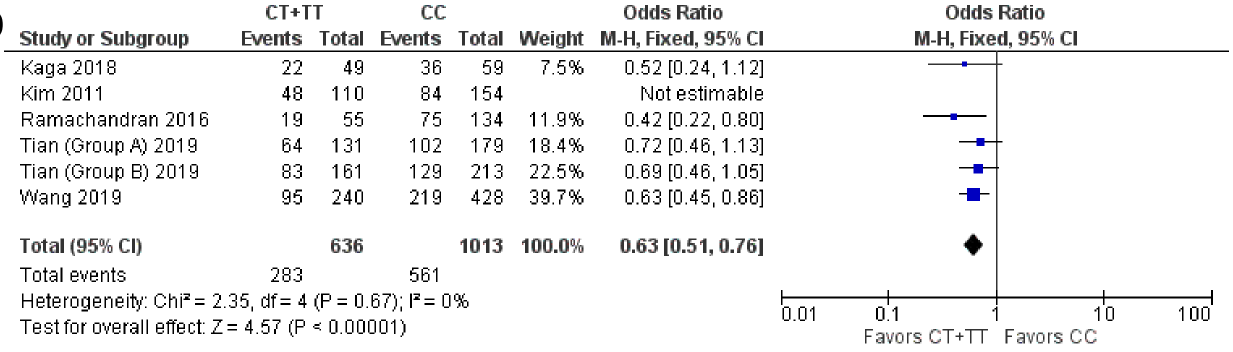

(I)

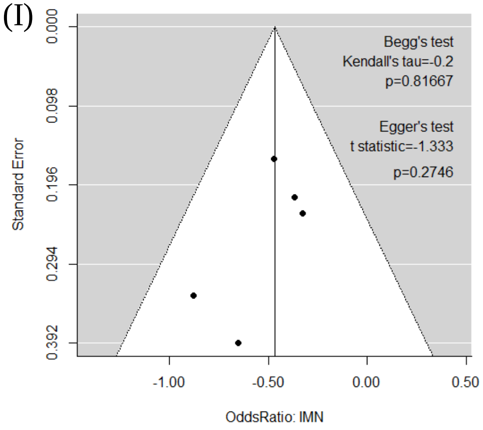

(E)

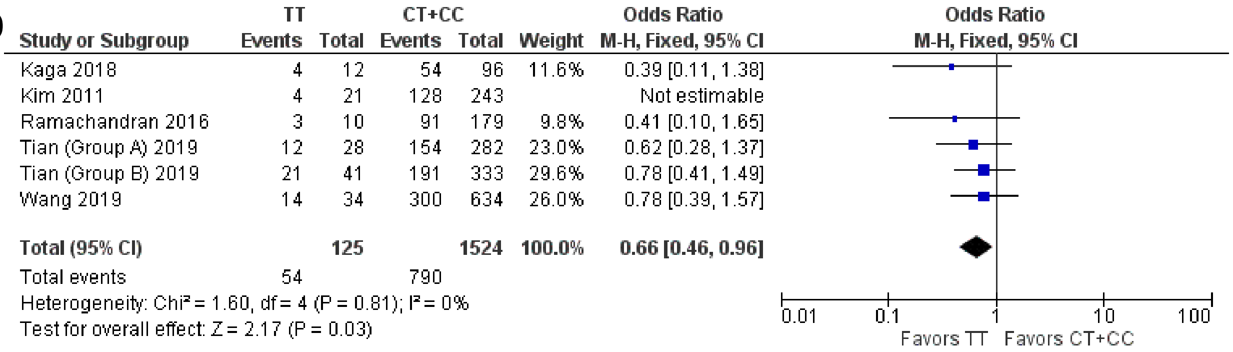

(J)

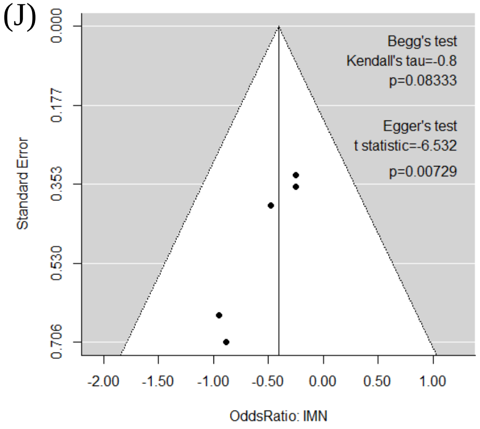

Supplementary Figure S1

Forest plot of the risk of IMN associated with rs3828323 under the (A) allelic model (T vs. C), (B) additive model (TT vs. CC), (C) additive model (CT vs. CC), (D) dominant model (CT+TT vs. CC), and (E) recessive model (TT vs. CT+CC). (F-J) Funnel plot, Begg's test and Egger's test for (A-E), respectively. Note that the log (OR) is plotted on the horizontal axis.

(A)

Study or Subgroup	C Events	C Total	G Events	G Total	Weight	Odds Ratio M-H, Random, 95% CI
Kaga 2018	28	81	88	135		Not estimable
Kim 2011	219	405	45	123	12.2%	2.04 [1.35, 3.09]
Liu 2010	41	97	217	373	12.0%	0.53 [0.33, 0.83]
Lv 2013	344	959	1880	3305	13.3%	0.42 [0.37, 0.49]
Ramachandran 2016	22	73	166	305	11.4%	0.36 [0.21, 0.63]
Saeed 2014	147	477	411	755	13.0%	0.37 [0.29, 0.47]
Tian (Group A) 2019	258	455	74	165	12.5%	1.61 [1.13, 2.30]
Tian (Group B) 2019	339	571	85	177	12.6%	1.58 [1.13, 2.22]
Wang 2019	96	308	532	1028	12.9%	0.42 [0.32, 0.55]
**Total (95% CI)**		**3345**		**6231**	**100.0%**	**0.71 [0.44, 1.13]**
Total events	1466		3410			

Heterogeneity: Tau² = 0.42; Chi² = 137.65, df = 7 (P < 0.00001); I² = 95%
Test for overall effect: Z = 1.44 (P = 0.15)

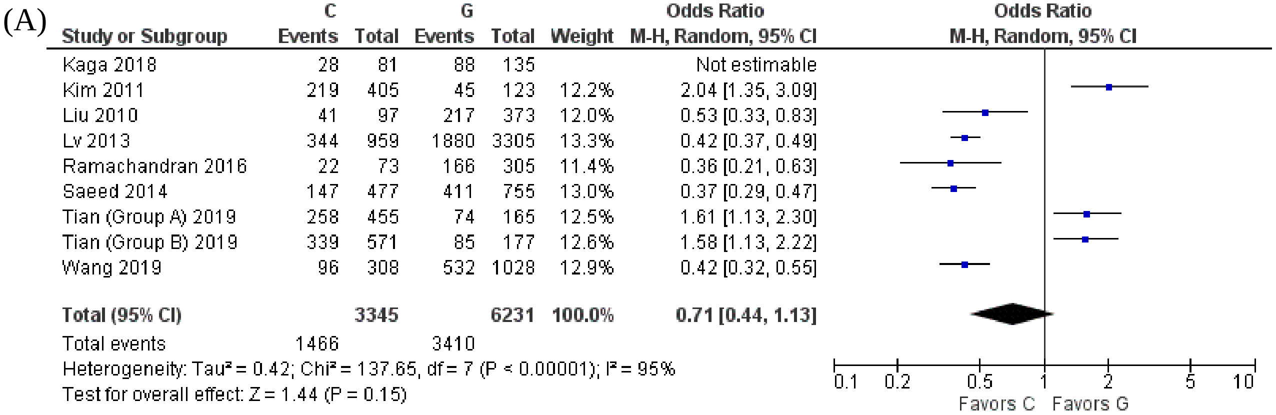

(F)

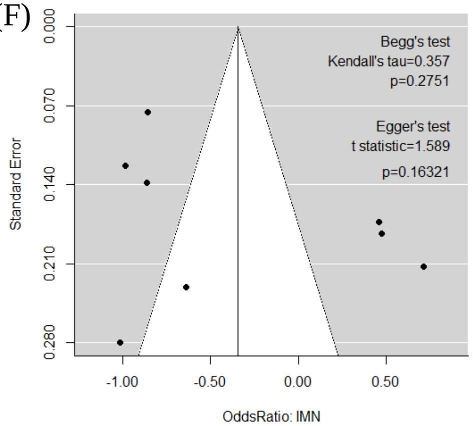

(B)

Study or Subgroup	CC Events	CC Total	GG Events	GG Total	Weight	Odds Ratio M-H, Random, 95% CI
Kaga 2018	5	24	35	51		Not estimable
Kim 2011	96	163	9	22	12.8%	2.07 [0.84, 5.12]
Liu 2010	2	10	90	148	9.5%	0.16 [0.03, 0.79]
Lv 2013	33	114	801	1287	14.8%	0.25 [0.16, 0.38]
Ramachandran 2016	2	7	74	123	9.1%	0.26 [0.05, 1.42]
Saeed 2014	28	110	160	249	14.5%	0.19 [0.12, 0.31]
Tian (Group A) 2019	101	169	9	24	12.9%	2.48 [1.03, 5.98]
Tian (Group B) 2019	137	217	10	20	12.8%	1.71 [0.68, 4.29]
Wang 2019	9	45	227	405	13.5%	0.20 [0.09, 0.42]
**Total (95% CI)**		**835**		**2278**	**100.0%**	**0.50 [0.23, 1.11]**
Total events	408		1380			

Heterogeneity: Tau² = 1.05; Chi² = 56.68, df = 7 (P < 0.00001); I² = 88%
Test for overall effect: Z = 1.70 (P = 0.09)

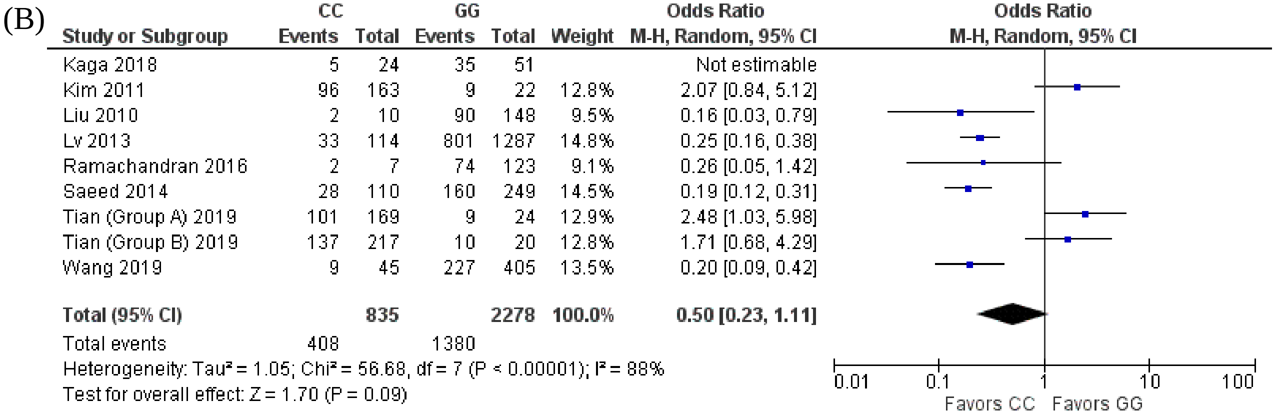

(G)

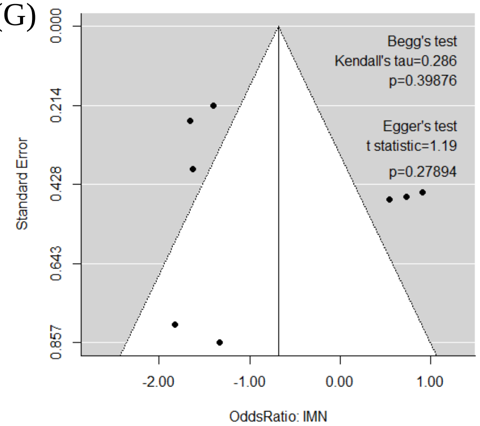

(C)

Study or Subgroup	CG Events	CG Total	GG Events	GG Total	Weight	Odds Ratio M-H, Random, 95% CI
Kaga 2018	18	33	35	51		Not estimable
Kim 2011	27	79	9	22	6.3%	0.75 [0.28, 1.98]
Liu 2010	37	77	90	148	12.5%	0.60 [0.34, 1.04]
Lv 2013	278	731	801	1287	21.9%	0.37 [0.31, 0.45]
Ramachandran 2016	18	59	74	123	10.4%	0.29 [0.15, 0.56]
Saeed 2014	91	257	160	249	17.3%	0.30 [0.21, 0.44]
Tian (Group A) 2019	56	117	9	24	7.0%	1.53 [0.62, 3.77]
Tian (Group B) 2019	65	137	10	20	6.6%	0.90 [0.35, 2.31]
Wang 2019	78	218	227	405	18.0%	0.44 [0.31, 0.61]
**Total (95% CI)**		**1675**		**2278**	**100.0%**	**0.47 [0.35, 0.62]**
Total events	650		1380			

Heterogeneity: Tau² = 0.09; Chi² = 18.81, df = 7 (P = 0.009); I² = 63%
Test for overall effect: Z = 5.26 (P < 0.00001)

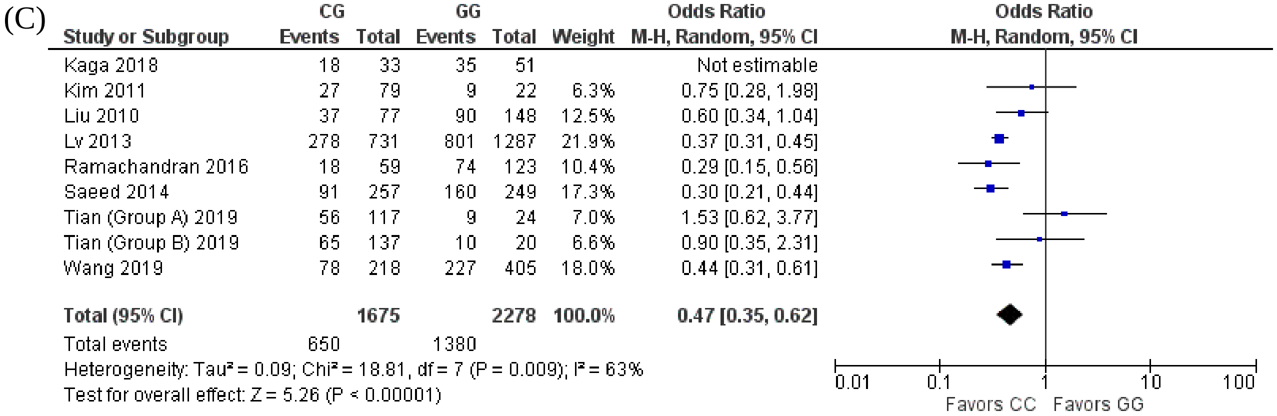

(H)

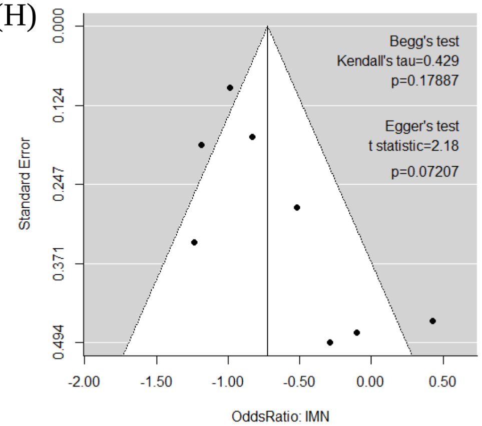

Supplementary Figure S2 (continued)

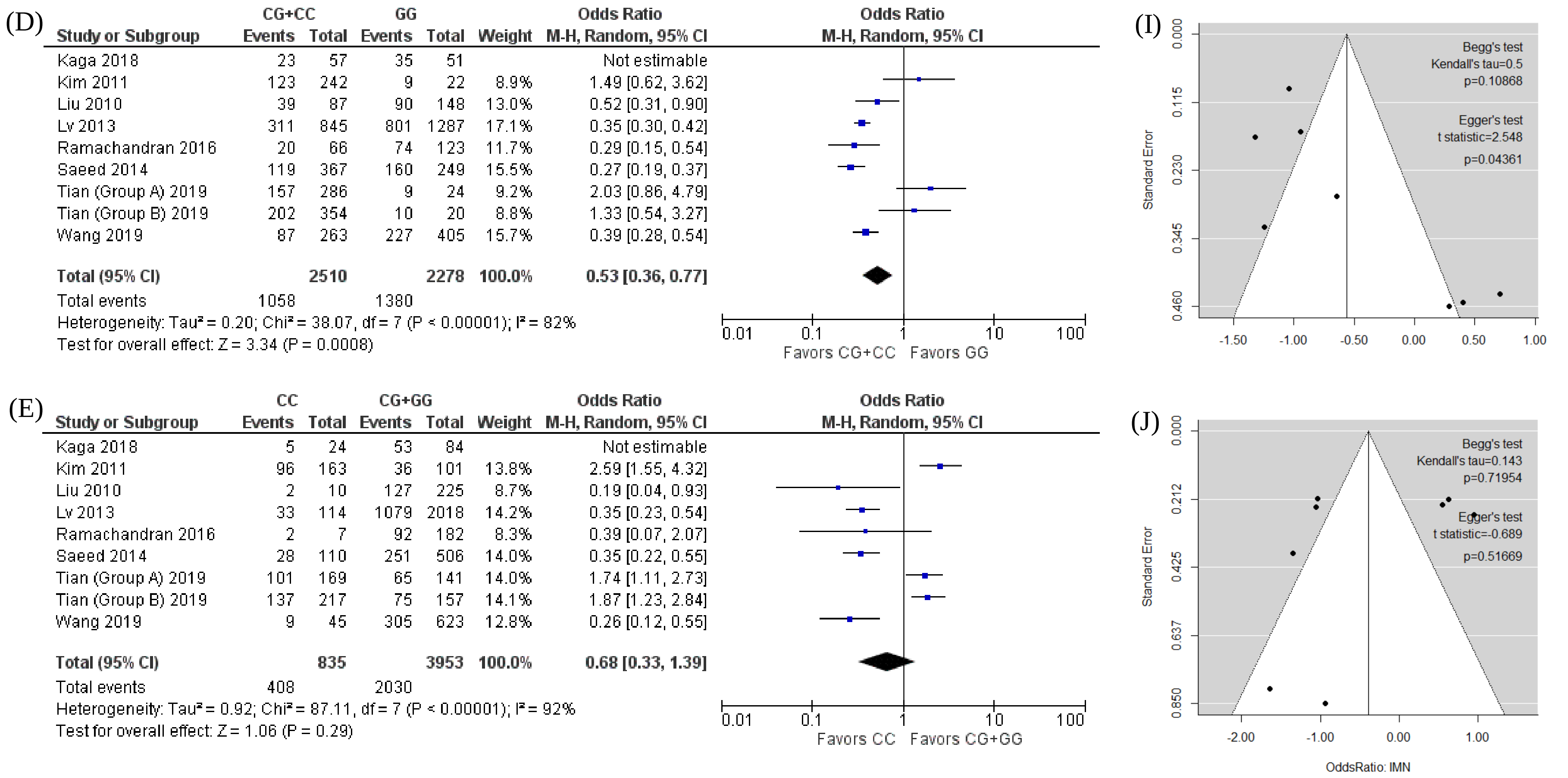

Supplementary Figure S2

Forest plot of the risk of IMN associated with rs35771982 under the (A) allelic model (C vs. G), (B) additive model (CC vs. GG), (C) additive model (CG vs. GG), (D) dominant model (CG+CC vs. GG), and (E) recessive model (CC vs. CG+GG). (F-J) Funnel plot, Begg's test and Egger's test for (A-E), respectively. Note that the log (OR) is plotted on the horizontal axis.

(A)

Study or Subgroup	C Events	C Total	T Events	T Total	Weight	Odds Ratio M-H, Random, 95% CI
Kaga 2018	32	86	84	130		Not estimable
Lv 2013	346	957	1878	3307	28.5%	0.43 [0.37, 0.50]
Ramachandran 2016	73	139	115	239	22.4%	1.19 [0.78, 1.81]
Tian (Group A) 2019	83	184	249	436	24.3%	0.62 [0.44, 0.87]
Tian (Group B) 2019	95	201	329	547	24.8%	0.59 [0.43, 0.82]
**Total (95% CI)**		**1481**		**4529**	**100.0%**	**0.64 [0.43, 0.96]**
Total events	597		2571			

Heterogeneity: $Tau^2 = 0.14$; $Chi^2 = 22.75$, df = 3 (P < 0.0001); $I^2 = 87\%$
Test for overall effect: Z = 2.18 (P = 0.03)

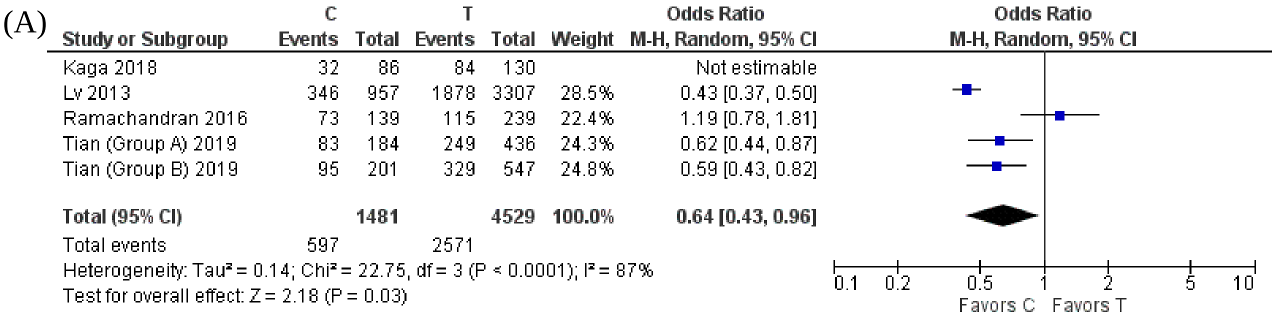

(B)

Study or Subgroup	CC Events	CC Total	TT Events	TT Total	Weight	Odds Ratio M-H, Random, 95% CI
Kaga 2018	5	24	31	46		Not estimable
Lv 2013	35	115	801	1290	33.5%	0.27 [0.18, 0.40]
Ramachandran 2016	11	25	32	75	19.2%	1.06 [0.42, 2.63]
Tian (Group A) 2019	12	34	95	160	22.7%	0.37 [0.17, 0.81]
Tian (Group B) 2019	17	38	134	211	24.7%	0.47 [0.23, 0.94]
**Total (95% CI)**		**212**		**1736**	**100.0%**	**0.43 [0.25, 0.74]**
Total events	75		1062			

Heterogeneity: $Tau^2 = 0.19$; $Chi^2 = 7.87$, df = 3 (P = 0.05); $I^2 = 62\%$
Test for overall effect: Z = 3.04 (P = 0.002)

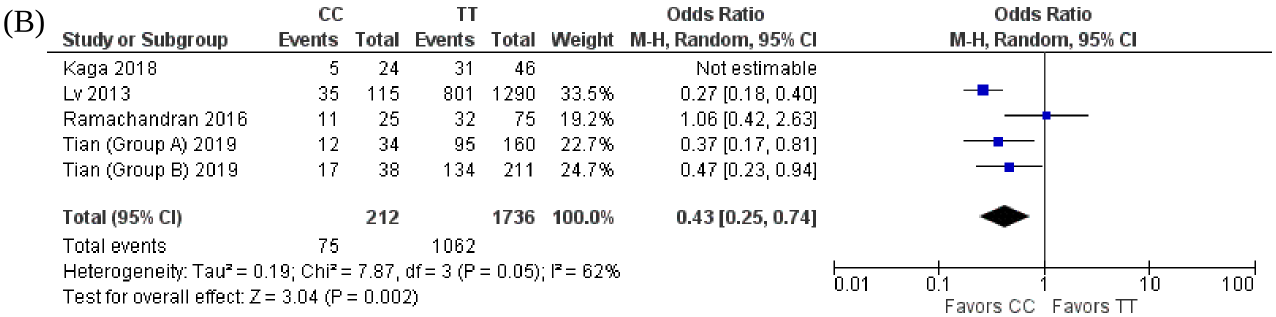

(C)

Study or Subgroup	CT Events	CT Total	TT Events	TT Total	Weight	Odds Ratio M-H, Random, 95% CI
Kaga 2018	22	38	31	46		Not estimable
Lv 2013	276	727	801	1290	28.2%	0.37 [0.31, 0.45]
Ramachandran 2016	51	89	32	75	22.2%	1.80 [0.97, 3.36]
Tian (Group A) 2019	59	116	95	160	24.5%	0.71 [0.44, 1.15]
Tian (Group B) 2019	61	125	134	211	25.0%	0.55 [0.35, 0.86]
**Total (95% CI)**		**1057**		**1736**	**100.0%**	**0.68 [0.37, 1.25]**
Total events	447		1062			

Heterogeneity: $Tau^2 = 0.33$; $Chi^2 = 26.98$, df = 3 (P < 0.00001); $I^2 = 89\%$
Test for overall effect: Z = 1.24 (P = 0.22)

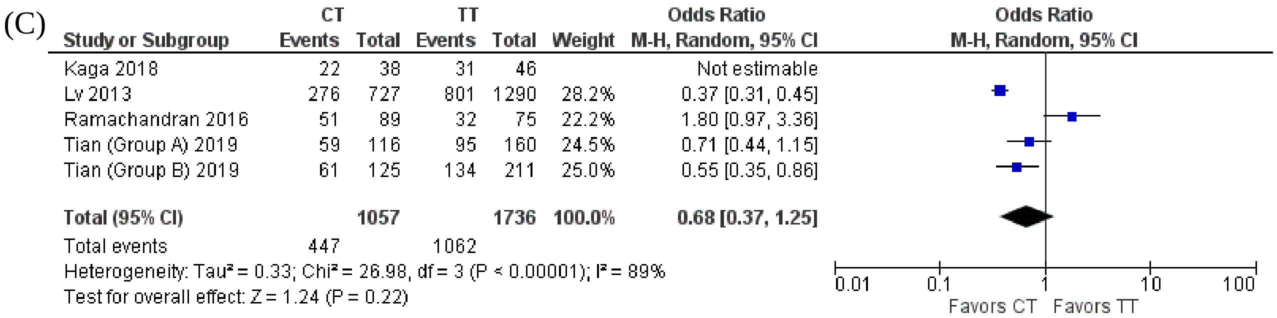

(F)

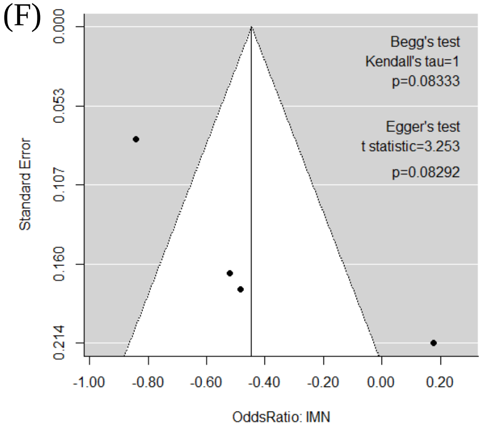

(G)

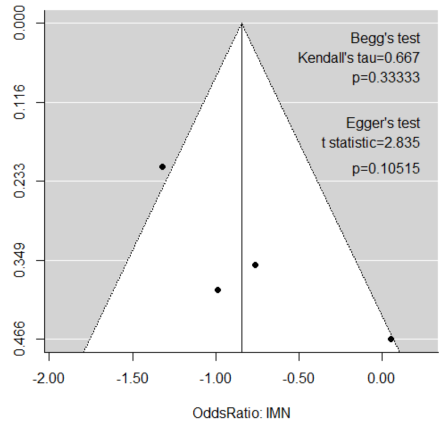

(H)

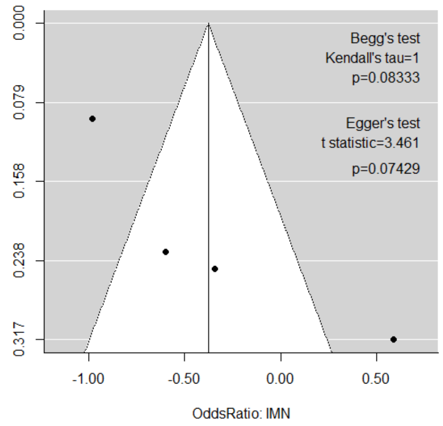

Supplementary Figure S3 (continued)

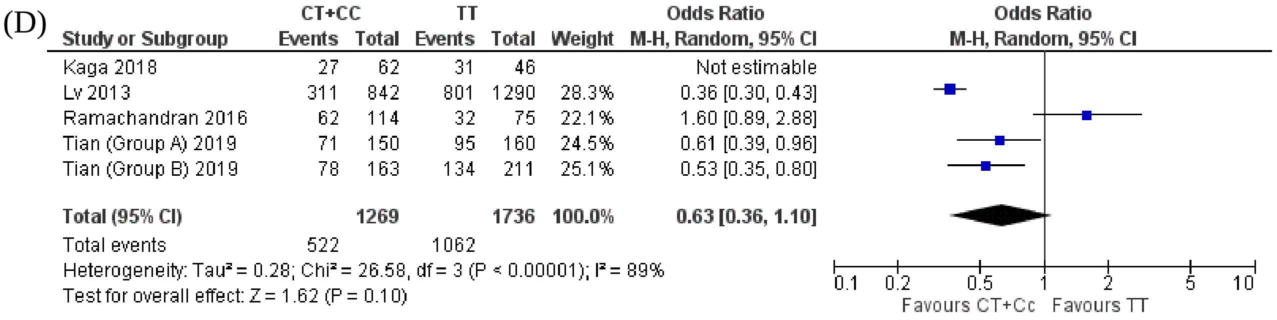

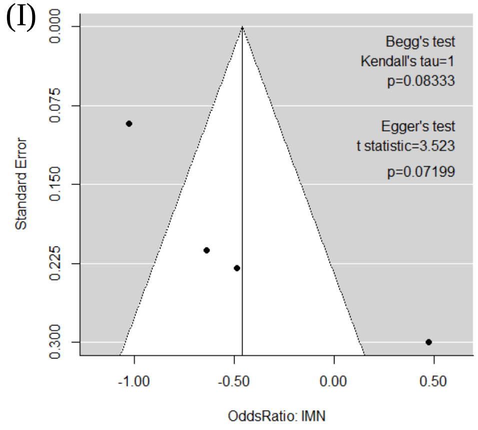

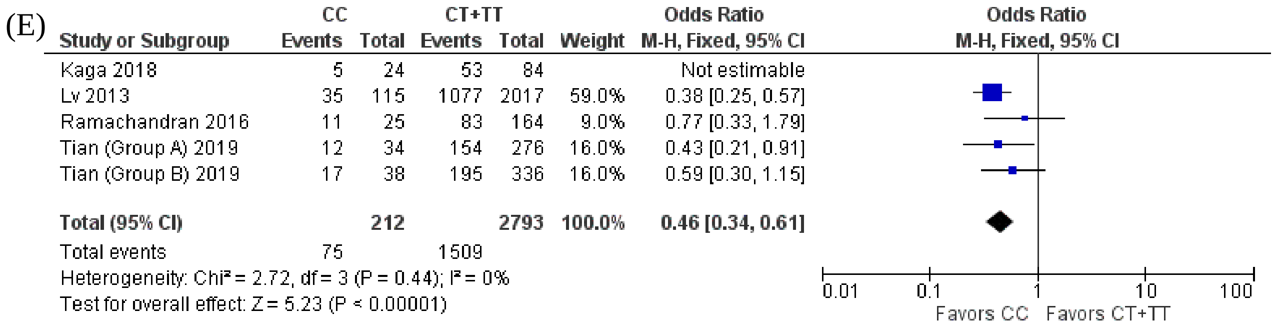

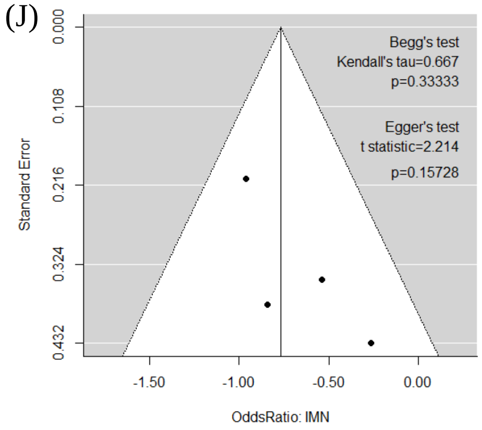

Supplementary Figure S3

Forest plot of the risk of IMN associated with rs3749117 under the (A) allelic model (C vs. T), (B) additive model (CC vs. TT), (C) additive model (CT vs. TT), (D) dominant model (CT+CC vs. TT), and (E) recessive model (CC vs. CT+TT). (F-J) Funnel plot, Begg's test and Egger's test for (A-E), respectively. Note that the log (OR) is plotted on the horizontal axis.

(A)

Study or Subgroup	T Events	T Total	C Events	C Total	Weight	Odds Ratio M-H, Fixed, 95% CI
Kaga 2018	32	73	84	143	17.7%	0.55 [0.31, 0.97]
Ramachandran 2016	22	81	166	297		Not estimable
Tian (Group A) 2019	94	190	238	430	40.8%	0.79 [0.56, 1.11]
Tian (Group B) 2019	94	179	330	569	41.5%	0.80 [0.57, 1.12]
Wang 2019	91	304	537	1032		Not estimable
**Total (95% CI)**		**442**		**1142**	**100.0%**	**0.75 [0.60, 0.94]**
Total events	220		652			

Heterogeneity: Chi² = 1.40, df = 2 (P = 0.50); I² = 0%
Test for overall effect: Z = 2.53 (P = 0.01)

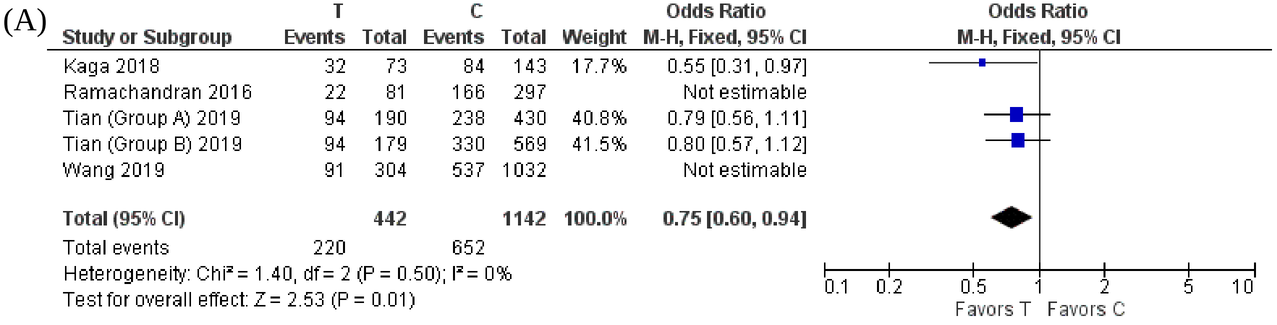

(B)

Study or Subgroup	TT Events	TT Total	CC Events	CC Total	Weight	Odds Ratio M-H, Fixed, 95% CI
Kaga 2018	3	9	29	44	19.6%	0.26 [0.06, 1.18]
Ramachandran 2016	8	27	80	135		Not estimable
Tian (Group A) 2019	14	31	86	151	47.9%	0.62 [0.29, 1.35]
Tian (Group B) 2019	12	22	130	217	32.5%	0.80 [0.33, 1.94]
Wang 2019	9	49	232	413		Not estimable
**Total (95% CI)**		**62**		**412**	**100.0%**	**0.61 [0.36, 1.04]**
Total events	29		245			

Heterogeneity: Chi² = 1.60, df = 2 (P = 0.45); I² = 0%
Test for overall effect: Z = 1.80 (P = 0.07)

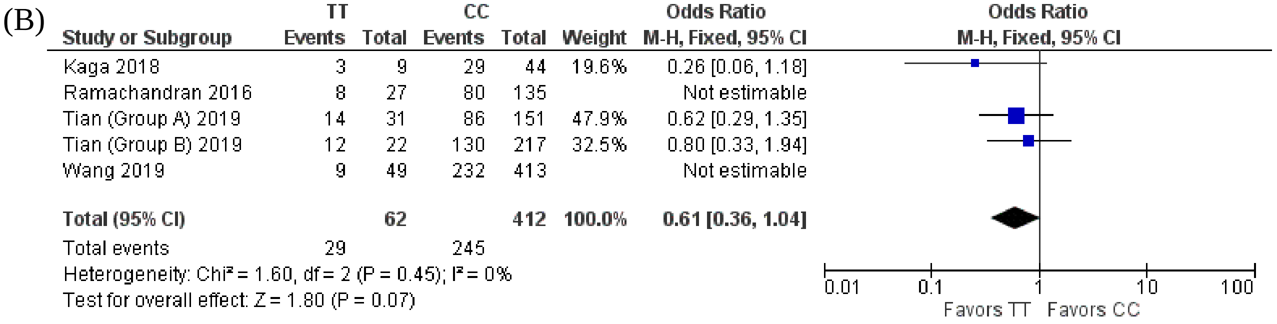

(C)

Study or Subgroup	CT Events	CT Total	CC Events	CC Total	Weight	Odds Ratio M-H, Fixed, 95% CI
Kaga 2018	26	55	29	44	16.5%	0.46 [0.20, 1.05]
Ramachandran 2016	6	27	80	135		Not estimable
Tian (Group A) 2019	66	128	86	151	37.0%	0.80 [0.50, 1.29]
Tian (Group B) 2019	70	135	130	217	46.5%	0.72 [0.47, 1.11]
Wang 2019	73	206	232	413		Not estimable
**Total (95% CI)**		**318**		**412**	**100.0%**	**0.71 [0.53, 0.95]**
Total events	162		245			

Heterogeneity: Chi² = 1.32, df = 2 (P = 0.52); I² = 0%
Test for overall effect: Z = 2.27 (P = 0.02)

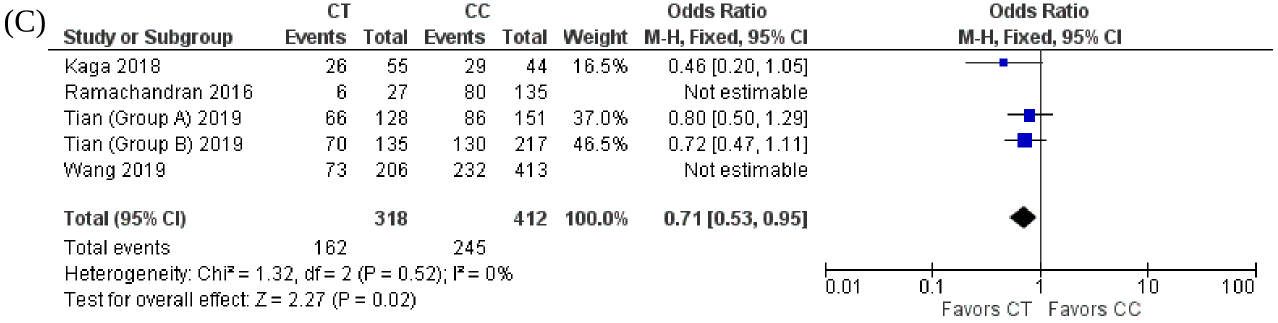

(F)

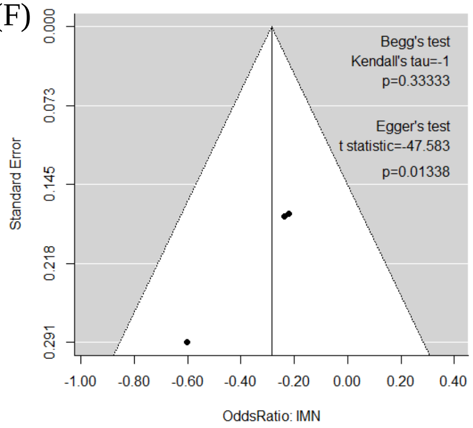

(G)

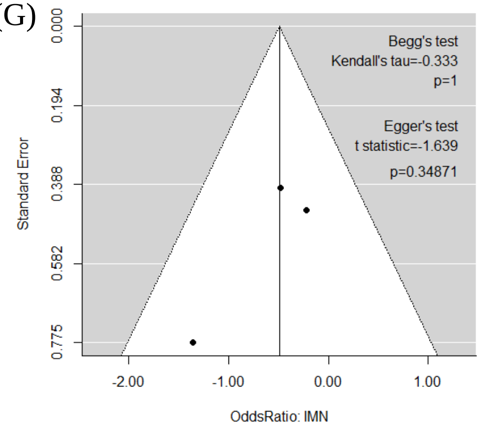

(H)

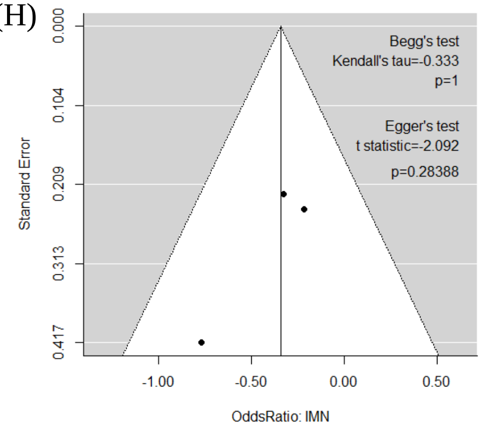

Supplementary Figure S4 (continued)

(D)
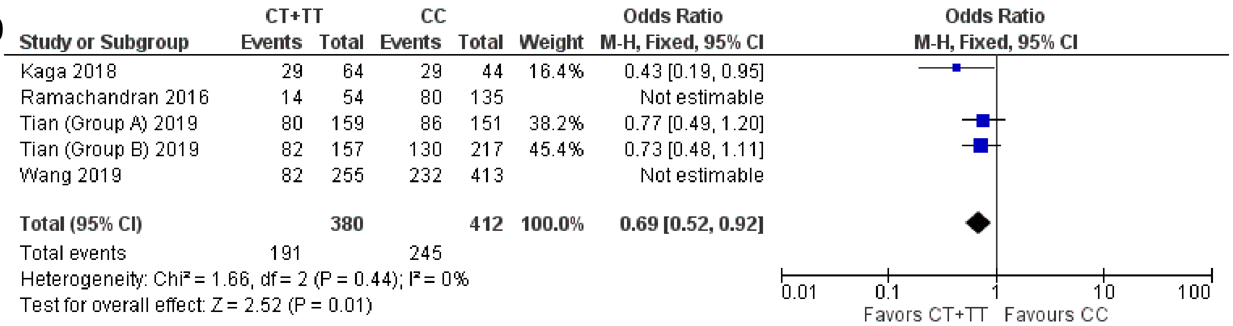

(I)
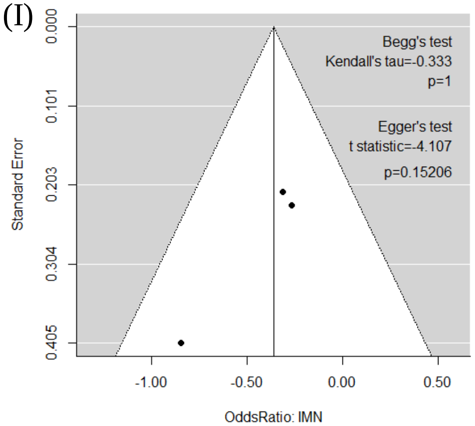

(E)
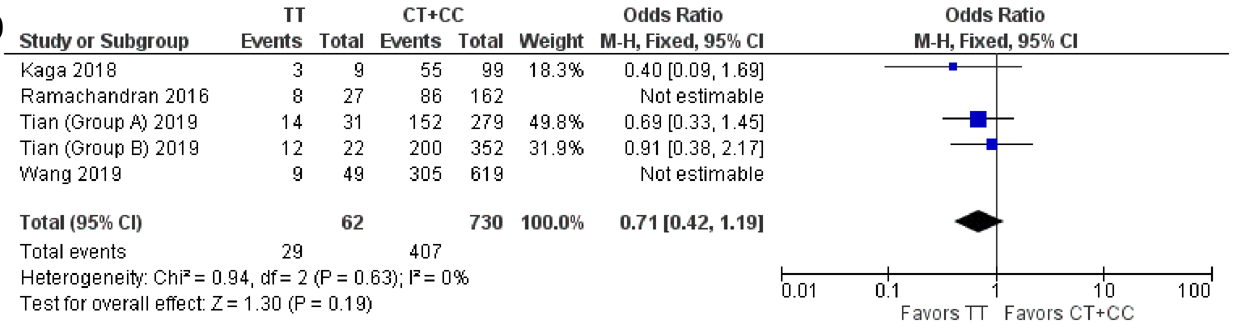

(J)
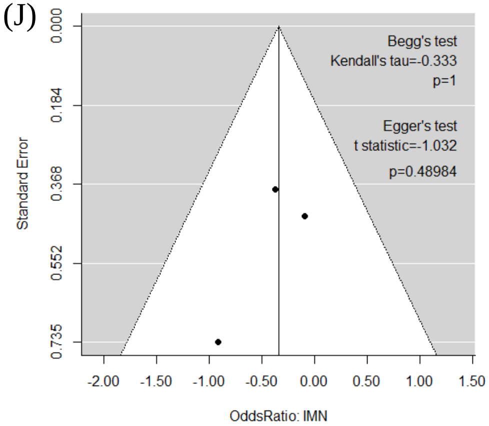

Supplementary Figure S4

Forest plot of the risk of IMN associated with rs3749119 under the (A) allelic model (T vs. C), (B) additive model (TT vs. CC), (C) additive model (CT vs. CC), (D) dominant model (CT+TT vs. CC), and (E) recessive model (TT vs. CT+CC). (F-J) Funnel plot, Begg's test and Egger's test for (A-E), respectively. Note that the log (OR) is plotted on the horizontal axis.
